# Integration Analysis of MicroRNA and mRNA Expression Profiles in Human Peripheral Blood Lymphocytes Cultured in Modeled Microgravity

**DOI:** 10.1155/2014/296747

**Published:** 2014-06-23

**Authors:** C. Girardi, C. De Pittà, S. Casara, E. Calura, C. Romualdi, L. Celotti, M. Mognato

**Affiliations:** ^1^Dipartimento di Biologia, Università degli Studi di Padova, Via U. Bassi 58/B, 35131 Padova, Italy; ^2^Laboratori Nazionali di Legnaro, INFN, Viale dell'Università 2, Legnaro, 35020 Padova, Italy

## Abstract

We analyzed miRNA and mRNA expression profiles in human peripheral blood lymphocytes (PBLs) incubated in microgravity condition, simulated by a ground-based rotating wall vessel (RWV) bioreactor. Our results show that 42 miRNAs were differentially expressed in MMG-incubated PBLs compared with 1 g incubated ones. Among these, miR-9-5p, miR-9-3p, miR-155-5p, miR-150-3p, and miR-378-3p were the most dysregulated. To improve the detection of functional miRNA-mRNA pairs, we performed gene expression profiles on the same samples assayed for miRNA profiling and we integrated miRNA and mRNA expression data. The functional classification of miRNA-correlated genes evidenced significant enrichment in the biological processes of immune/inflammatory response, signal transduction, regulation of response to stress, regulation of programmed cell death, and regulation of cell proliferation. We identified the correlation of miR-9-3p, miR-155-5p, miR-150-3p, and miR-378-3p expression with that of genes involved in immune/inflammatory response (e.g., IFNG and IL17F), apoptosis (e.g., PDCD4 and PTEN), and cell proliferation (e.g., NKX3-1 and GADD45A). Experimental assays of cell viability and apoptosis induction validated the results obtained by bioinformatics analyses demonstrating that in human PBLs the exposure to reduced gravitational force increases the frequency of apoptosis and decreases cell proliferation.

## 1. Introduction

Exposure to spaceflight environment is known to cause in humans many adverse physiological changes, including skeletal muscle atrophy [1–3], cardiovascular and microvascular disorders [4–6], bone deterioration [7, 8], and impaired immune system function [9, 10]. Immune system dysfunction due to exposure to microgravity has been documented as well in terms of reduced activation/proliferation, altered cytokine production, and altered signal transduction [11, 12]. Alterations in global gene expression patterns have been also observed in space-flown human cells, involving mainly genes of immune system activation [13, 14], cytoskeleton [15], and cell cycle [16, 17]. However, due to the difficulty and limitations of performing experiments in the real microgravity of space, many investigations have been conducted under simulated microgravity conditions, in which cells are cultured in ground-based machines, such as clinostats and rotating wall vessel bioreactors that generate a residual 10^−3^–10^−6 ^g force that approximates microgravity [5, 18–24]. The results indicate that, similar to space microgravity, simulated microgravity affects both cell structure and function, as well as gene expression, in mammalian cells [14, 19, 25], in bacteria [26, 27], or in other living organisms [28–30].

Since molecular changes at the gene level may compromise cell function, it is important to understand the cellular response to reduced gravity at the molecular level. For this purpose, a class of noncoding RNAs, called microRNAs (miRNAs), plays a key role. miRNAs are a large family of small RNAs of 18–24 nucleotides that are involved in posttranscriptional regulation of gene expression by interacting with 3′-untranslated regions (UTR) of target genes. The regulatory process is complex and occurs posttranscriptionally through miRNA interaction with a target site in the mRNA that has partial or complete complementarity to the miRNA. The binding of miRNAs to complementary sequence of their target mRNAs may repress translation or induce degradation of mRNAs [31]. Recently, the destabilization of target mRNAs, instead of translational repression, has been shown to be the predominant mechanism for reduced protein output [32]. Less often, dsRNA formed by miRNA target complexes can target gene promoters and actually enhance transcription of target genes, sometimes termed RNAa (RNA activation) [33]. A single miRNA may have broad effects on gene expression networks, such as regulating cell lineage specificity, cellular functions, or stress response. Besides a physiological role of miRNAs in a variety of important biological processes including differentiation, apoptosis [34], and fat metabolism [35], the miRNA-mediated gene regulation operates also during viral infection [36], stress response pathway [37], and pathological processes, such as tumorigenesis [38–42].

The present study is addressed to identify alterations in miRNA profiles of human peripheral blood lymphocytes (PBLs) incubated in modeled microgravity (MMG) with respect to those incubated in gravity 1 g. To simulate microgravity, we used a specialized bioreactor developed at the NASA-Johnson Space Center (Houston, TX, USA), the rotating wall vessel, which represents a valid ground model to simulate, as far as possible, a condition of reduced gravity. To identify miRNA-correlated genes whose expression level was significantly altered as a function of MMG, we performed gene expression profiling on the same PBL samples assayed for miRNA profiling and we integrated microRNAome and transcriptome by using MAGIA^2^ [43], a web tool for the integrative analysis of miRNA and genes expression data incorporating transcriptional regulation. A group of miRNA-mRNA pairs related to immunity, cell proliferation, and apoptosis was identified in PBLs incubated in MMG. The differences between MMG and 1 g on correlated miRNA-mRNA pairs involved in cell proliferation and apoptosis were investigated by* in vitro *assays of clonogenic ability and apoptosis induction in PBLs incubated in MMG with respect to those incubated in 1 g conditions.

## 2. Materials and Methods

### 2.1. Lymphocytes Isolation and Microgravity Simulation

Human peripheral blood lymphocytes (PBLs) were obtained from freshly collected “buffy coats” from blood samples of twelve healthy anonymous donors at the Blood Centre of the City Hospital of Padova (Italy). This study obtained the Ethics Approval from the Transfusion Medicine (TM) Ethics Committee of Blood Centre of the City Hospital of Padova. PBLs were isolated by separation on Biocoll density gradient (BIOCHROM, Berlin, Germany). After isolation, PBLs were preincubated overnight at a concentration of 3 × 10^6^/mL in basal medium RPMI 1640 containing GlutaMAX I (Invitrogen Life Technologies, Carlsbad, CA, USA), 124 U/mL penicillin, 63 *μ*g/mL streptomycin sulfate, and 10% fetal bovine serum (FBS, BIOCHROM, Berlin, Germany). After the overnight incubation, PBLs, consisting of peripheral mononuclear cells depleted of monocytes, were suspended at 1 × 10^6^/mL in basal medium and subjected to modeled microgravity, simulated by the rotating wall vessel (RWV) bioreactor (Synthecon, Cellon), placed inside a humidified incubator, vertically rotating at 23 rpm [44]. In the rotating system, the gravity is balanced by equal and opposite mechanical forces (centrifugal, Coriolis, and shear components), and the gravitational vector is reduced to about 10^−3 ^g. In these conditions, single cells are nearly always in suspension, rotating in quasi-stationary manner with the fluid, in a low-shear culture environment [19, 45]. Ground-based (1 g) PBLs were kept at the same cell density in 75 cm^2^ flasks (FALCON) in the same medium. After 24, 48, or 72 h of incubation in MMG and 1 g PBLs were activated to enter cell cycle to measure cell proliferation by incubation in culture medium (CM) containing phytohaemagglutinin (PHA, BIOCHROM, Berlin, Germany) and interleukin 2 (IL2, Chiron Siena, Italy) as stimulating factors [46].

### 2.2. Total RNA Isolation

Total RNA was isolated from 10^7^ PBLs at the end of 24 h incubation in MMG and 1 g by using Trizol Reagent (Invitrogen Life Technologies, Carlsbad, CA, USA), according to the manufacturer's protocol. Total RNA was quantified using the ND-1000 spectrophotometer (Nanodrop, Wilmington, DE, USA) and RNA integrity and the content of miRNAs were assessed by capillary electrophoresis using the Agilent Bioanalyzer 2100, as previously described [47]. Only total RNA samples with RNA integrity number (RIN) values ≥6 and with miRNA <20% were used for microarray analysis.

### 2.3. miRNA and Gene Expression Profiling

MicroRNAs profiling was carried out in PBL samples incubated in MMG versus 1 g. Analyses were performed by using the “Human miRNA Microarray kit (V2)” (Agilent Technologies) that allows the detection of 723 known human (miRBase v.10.1) and 76 human viral miRNAs. Total RNA (200 ng) was labeled with pCp Cy3, according to the Agilent protocol and unincorporated dyes were removed with MicroBioSpin6 columns (BioRad) [48]. Probes hybridization and slides washing were performed as previously reported [47]. Agilent Feature Extraction software version 10.5.1.1 was used for image analysis.

Gene expression profiling was carried out in MMG-incubated PBLs versus 1 g incubated PBLs on total RNA extracted from the same PBL samples assayed for miRNA profiling. We used the “Whole Human Genome Oligo Microarray” (Agilent), consisting of ~41.000 (60-mer) oligonucleotide probes, which span conserved exons across the transcripts of the targeted full-length genes. 800 ng of total RNA was labeled with “Agilent One-Color Microarray-Based Gene Expression protocol” according to the manufacturer's instructions. The method uses T7 RNA polymerase, which simultaneously amplifies target material and incorporates cyanine 3-labeled CTP. The Cy3-labeled cRNAs were purified using Qiagen's RNeasy mini spin columns (Qiagen) and quantified using the ND-1000 spectrophotometer (Nanodrop, Wilmington, DE, USA). Probes hybridization and slides washing were performed as previously reported [47]. Slides were scanned on an Agilent Microarray Scanner System (model G2565CA) and Agilent Feature Extraction software version 10.5.1.1 was used for image analysis. Raw data are available on the Gene Expression Omnibus (GEO) website (http://www.ncbi.nlm.nih.gov/geo/) using SuperSeries accession number GSE57418 that groups microRNA (GSE57400) and mRNA expression profiles (GSE57408).

### 2.4. Statistical Analysis of miRNA and Gene Expression Data

Interarray normalization of expression levels was performed with cyclic Lowess for miRNA experiments and with quantile for gene expression profiling [49] to correct possible experimental distortions. Normalization function was applied to expression data of all experiments and then values of spot replicates within arrays were averaged. The modalities of spot quality measures and hybridization are reported previously [47]. The identification of differentially expressed genes and miRNAs was performed with one- and two-class Significance Analysis of Microarray (SAM) program [50] with default settings. The expression level of each miRNA and mRNA was calculated as the log2 (MMG/1 g) PBLs of the same donor. Pathway analysis on differentially expressed genes has been performed using Graphite web [51], hypergeometric test on Reactome Pathways, considering significant those categories with FDR < 0.1.

### 2.5. Identification of miRNA Target Genes and Correlation Analysis of miRNA and mRNA Expression Data

To predict miRNA targets, we performed a computational analysis integrating mRNA and miRNA expression measurements from the same donor using MAGIA^2^ web tool [43], freely available at http://gencomp.bio.unipd.it/magia2/. We used http://www.microrna.org/ predictions and Pearson correlation (*r* > 0.4) to estimate the degree of correlation between any putative pairs of miRNA and mRNA [52, 53]. To identify the biological processes most involved in target prediction, we have performed an enrichment analysis on Gene Ontology (GO) using DAVID web tool v6.7 [54] considering significant those categories with FDR < 0.2.

Intraclass analyses have been performed considering MMG and 1 g samples separately. In order to have comparable results, the intraclass analysis has been performed using MAGIA^2^ software with the same parameters, predictors, and cutoff described for the previous analysis. Specific interactions for MMG and 1 g networks have been identified and a GO enrichment analysis (FDR < 0.2) was performed separately on the nodes belonging to the specific MMG and 1 g networks using DAVID web tool.

### 2.6. Quantitative Real-Time PCR (qRT-PCR) Assay

In order to verify the expression data generated by miRNA and mRNA microarrays, we performed qRT-PCR experiments for miRNAs and genes which showed significant expression changes in MMG. The following miRNAs were subjected to the RT-qPCR validation: miR-9-5p, miR-378a, miR-155-5p, and miR-150-3p. Reverse transcription of 10 ng of total RNA with primers corresponding to each miRNA and to U48 small nuclear RNA (RNU48) as endogenous control was performed as directed by the protocol of the two-step TaqMan MicroRNA Assay kit (Applied Biosystems, Foster City, CA, USA) that incorporates a target-specific stem-loop reverse transcription primer to provide specificity for the mature miRNA target. For the PCR reaction, 1 *μ*L of the RT reaction was combined with 0.5 *μ*L of TaqMan MicroRNA Assay 20x and 5 *μ*L of TaqMan Universal PCR Master Mix in a 10 *μ*L final volume. The reactions were incubated in a Mastercycler EP gradient *S* (Eppendorf) in 0.2 mL PCR tubes for 30 min at 16°C and 30 min at 42°C, followed by 5 min at 85°C, and then held at 4°C. The resulting cDNA was quantitatively amplified in 40 cycles on an ABI 7500 Real-Time PCR System, using TaqMan Universal PCR Master Mix and TaqMan MicroRNA Assays.

For mRNA detection, 1 *μ*g of total RNA was retrotranscribed with ImProm-II Reverse Transcription System (Promega). qRT-PCR was performed with the GoTaq qPCR Master Mix (Promega) and gene-specific primers for IFNG, IL17F, TLR4, HLA-DRB1, and BCL6 genes and for GAPDH as reference. qRT-PCR reactions were performed in quadruplicates, in PBL samples from 6 to 8 donors. Real-time PCR was performed using an Applied Biosystems 7500 Fast Real-Time PCR System with cycling conditions of 95°C for 10 min followed by 95°C for 15 sec and 60°C for 60 sec, 45 cycles in total. The relative expression levels of miRNAs and mRNAs between samples were calculated using the comparative delta CT (threshold cycle number) method (2^−ΔΔCT^) implemented in the 7500 Real-Time PCR System software [55]. Primers' pairs used are as follows: GAPDH (glyceraldehyde-3-phosphate dehydrogenase): fw 5′-TCCTCTGACTTCAACAGCGA-3′; rev 5′-GGGTCTTACTCCTTGGAGGC-3′; IFNG (interferon gamma): fw 5′-GGCATTTTGAAGAATTGGAAAG-3′; rev 5′-TTTGGATGCTCTGGTCATCTT-3′; IL17F (interleukin 17): fw 5′-GGCATCATCAATGAAAACCA-3′; rev 5′-TGGGGTCCCAAGTGACAG-3′; TLR4 (Toll-like receptor 4): fw 5′-CCTGCGTGAGACCAGAAAG-3′; rev 5′-TTCAGCTCCATGCATTGATAA-3′; HLA-DRB1 (major histocompatibility complex, class II, DR beta 1): fw 5′-ACAACTACGGGGTTGTGGAG-3′; rev 5′-GCTGCCTGGATAGAAACCAC-3′; BCL6 (B-cell CLL/lymphoma 6): fw 5′-CGAATCCACACAGGAGAGAAA-3′; rev 5′-ACGCGGTATTGCACCTTG-3′.

### 2.7. Cell Proliferation and Apoptosis Induction

Cell viability was determined by the T-cell cloning assay [44, 46] at the end of 24 h, 48 h, and 72 h incubation in MMG and 1 g. Briefly, four 96-well U-bottomed microtiter plates with two viable lymphocytes/well were seeded in medium CM in the presence of 1 × 10^4^ feeder cells/well (TK6 lymphoblastoid cells lethally irradiated with 40 Gy of *γ*-rays). Two weeks later, the plates were scored for growing colonies to calculate the cloning efficiency (CE) from the proportion of negative wells assuming a Poisson distribution (CE = −In* P*_0_/*N*, where* P*_0_ is the fraction of wells without cells and* N* is the number of cells seeded into wells) [56].

Apoptotic index was determined in PBLs incubated for 24 and 48 h in MMG and in parallel in 1 g. For detection of apoptotic morphology, PBLs were fixed and stained with 2 *μ*g/mL 4,6-diamino-2-phenylindol (DAPI, Roche), in an antifade solution (Vectashield, Vector Lab) as previously described [57]. At least 2000 cells were scored for each time-point by fluorescence microscopy (1000x magnification). The activation of caspase-3 was measured by the caspase-3 fluorescent assay kit (Clontech BD Biosciences) at the end of incubation in 1 g or MMG, as previously described [58]. The fluorescent emission at 505 nm (excitation at 400 nm) of cleaved 7-amino-4-trifluoromethyl coumarin (AFC) was measured with a PerkinElmer LS-50 B spectrofluorimeter.

## 3. Results

### 3.1. Identification of miRNAs Affected by Microgravity

**Figure 1:**
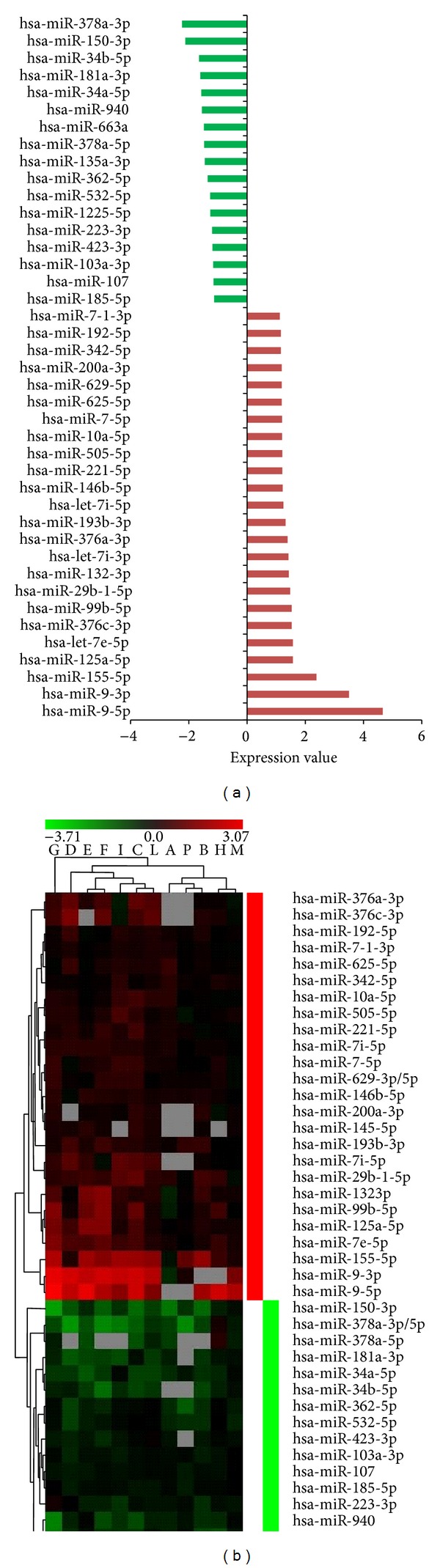
Differentially expressed miRNAs in human PBLs incubated in MMG. (a) The expression level of each miRNA, indicated as fold change, is the mean of the expression values obtained from the transformed log2 ratio (MMG/1 g). (b) Dendrogram of miRNAs differentially expressed in MMG. The range of expression value is from −3.7 (green, downregulation) to 3.07 (red, upregulation). Grey boxes correspond to not available (N/A) fluorescent signal from the microarray platform.

miRNA expression profiling was performed on total RNA extracted from PBLs of twelve healthy donors at the end of 24 h incubation time in MMG and in 1 g conditions. By comparing the expression profile of MMG-incubated versus 1 g incubated PBLs of the same donor, we found 42 differentially expressed miRNAs, 25 upregulated and 17 downregulated, for which raw data and means of miRNA expression values are available at Supplementary Table S1 (see Supplementary Table S1 in the Supplementary Material available online at http://dx.doi.org/10.1155/2014/296747). miR-9-5p, miR-9-3p, and miR-155-5p were the most upregulated (4.6-, 3.5-, and 2.4-fold, resp.), whereas miR-378a and miR-150-3p were the most downregulated (~2-fold) (Figure 1).

### 3.2. Effect of Microgravity on Gene Expression Profile

**Figure 2:**
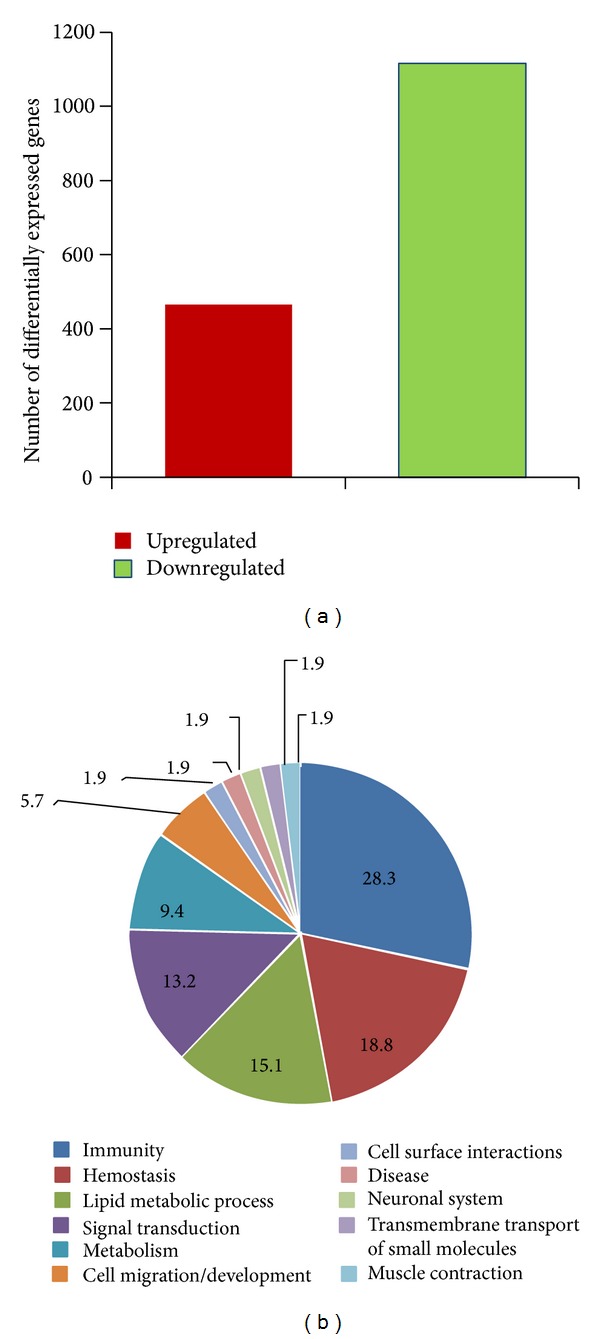
Results of gene expression analysis. Differentially expressed genes (a) and pie chart of biological process (%) containing pathways significantly enriched in PBLs incubated in MMG (b).

Gene expression analysis was performed in PBLs incubated for 24 h in MMG and in 1 g. By comparing the expression profiles of MMG-incubated and 1 g incubated PBLs, we identified 1581 differentially expressed genes in MMG versus 1 g, among which 465 (29%) genes were upregulated whereas 1116 (71%) genes were downregulated (Figure 2(a) and Supplementary Table S2). By selecting a 2-fold cut-off threshold, we identified 312 (19.7%) genes in MMG; 157 genes (10%) showed alterations in expression level with a fold change greater than 4.0, and, among these, 20 genes showed a fold change ≥16.0 (Supplementary Table S3). To identify sets of genes with expression changes in MMG condition, we used Graphite [51], a novel web tool for topological-based pathway analyses based on high-throughput gene expression data analyses. Pathway analysis on differentially expressed genes has been performed by using hypergeometric test on Reactome Pathways as implemented in Graphite web, considering significant those categories with FDR < 0.1. We evidenced biological pathways significantly enriched in MMG: 15 (28.3%) were related to immunity, 10 (18.8%) to hemostasis, 7 (13.2%) to lipid metabolic process and signal transduction, 5 (9.4%) to metabolism, 3 (5.7%) to cell migration/development, and 1 (1.9%) tocell surface interactions, disease, neuronal system, transmembrane transport of small molecules, and muscle contraction (Figure 2(b)). The list of pathways is reported in Supplementary Table S4.

**Figure 3:**
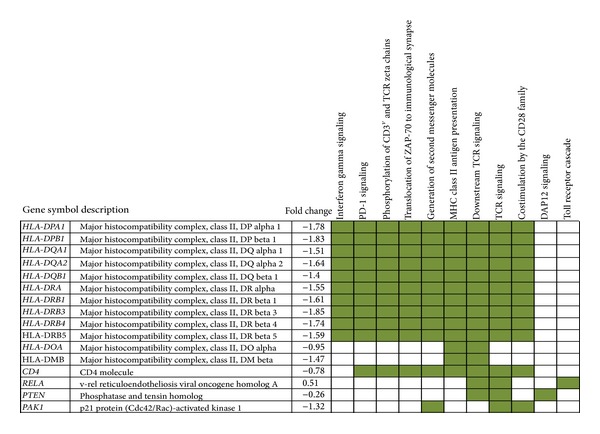
Differentially expressed genes common to immune-related pathways identified by Reactome database in PBLs incubated in MMG. The expression value of each gene, indicated as fold change, is the mean of expression levels calculated as the log2 ratio (MMG/1 g) on PBL samples (see Supplementary Table S2).

Among the immune pathways significantly enriched in MMG, those of “MHC class II antigen presentation,” “Toll Receptor Cascades,” and “DAP12 signaling” showed the highest number of differentially expressed genes (30, 20, and 20 genes, resp.). Also the “interferon gamma signaling” pathway was significantly enriched in MMG, with 13 differentially expressed genes. Ten genes codifying for proteins of MHC class II (HLA-DRA, HLA-DRB1, HLA-DRB3, HLA-DRB4, HLA-DRB5, HLA-DQA1, HLA-DQA2, HLA-DPA1, HLA-DPB1, and HLA-DQB1) were common to nine pathways (Figure 3). CD4, codifying for a membrane glycoprotein of T lymphocytes that interacts with the major histocompatibility complex class II antigens, was common to eight immune pathways; RELA, PTEN, and PAK1 were included in three pathways, whereas HLA-DOA and HLA-DMB were included in two pathways.

### 3.3. Target Prediction and Integration Analysis of miRNA and mRNA Expression Profiles

We examined the regulatory effects of miRNAs on global gene expression under modeled microgravity (MMG) condition in comparison with ground gravity (1 g). To predict the target genes of differentially expressed miRNAs in MMG, we performed a computational analysis using TargetScan tool, which predicts biological targets of miRNAs by searching for the presence of conserved 8 mer and 7 mer sites that match the seed region of each miRNA [59]. However, all available software for target prediction is characterized by a large fraction of false positive; thus, the integration of target predictions with miRNA and gene target expression profiles has been proposed to refine miRNA-mRNA interactions. The correlation analyses on the differentially expressed miRNAs and mRNAs were carried out with MAGIA^2^ software [43], by microRNA Pearson prediction analysis, which allowed the identification of miRNA-mRNA interactions (Supplementary Table S5). To discover functional relationships between miRNAs and the transcriptome and uncover the gene pathways that are regulated by miRNAs in MMG, we performed Gene Ontology (GO) analysis using DAVID [54].

In our analysis, we used high classification stringency and considered only GO terms that have *P* < 0.1 after permutation corrections (Benjamini) (Table 1). Several GO terms belonged to immune system function (i.e., “innate immune response,” “inflammatory response,” “regulation of cytokine production,” “positive regulation of immune system process,” and “response to bacterium”), in accordance with the results of pathway analysis on transcriptome (see Supplementary Table S4). GO terms of “cell development,” “regulation of cell differentiation,” “regulation of cell communication,” “cell motility,” and “cell migration” were significantly enriched in MMG, together with the category “organ development.” In addition, the biological categories of “regulation of signal transduction,” “regulation of response to stress,” “regulation of cell death,” and “regulation of cell proliferation” were enriched in PBLs incubated in MMG.

**Figure 4:**
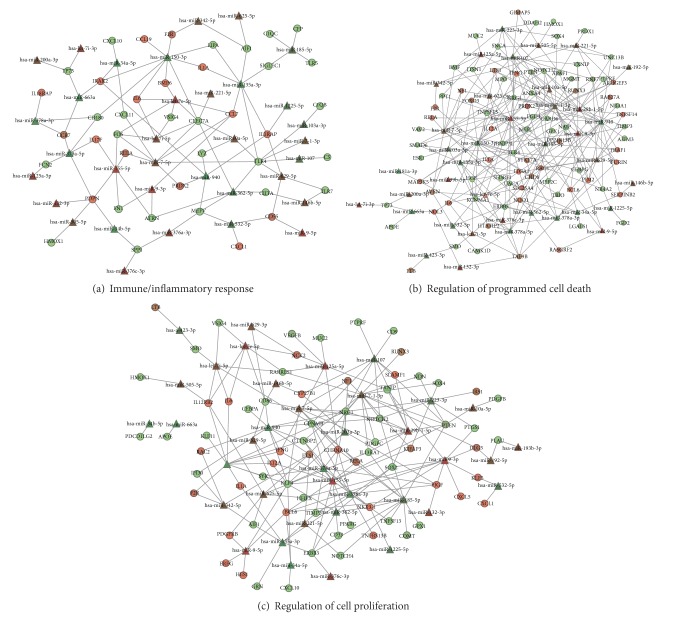
Network analysis on correlated miRNA-mRNA pairs in PBLs incubated in MMG. Network analyses were performed by MAGIA^2^ software using miRNAs correlating both positively and negatively with transcripts involved in immune/inflammatory response (a), in regulation of programmed cell death (b), and in regulation of cell proliferation (c). Circles represent transcripts and triangles represent miRNAs.

To determine whether different miRNAs within a GO category interact with the same target genes, we performed network analysis using MAGIA^2^ [43], a software platform for the visualization of complex miRNA-mRNA interactions. We focused on miRNAs that correlated both positively and negatively with the GO categories of immune/inflammatory response, regulation of programmed cell death, and regulation of cell proliferation. As shown in Figure 4, most transcripts are associated with more than one miRNA, as in the case of TLR4 transcript, correlated with eight different miRNAs (miR-10a-5p, miR-7-5p, miR-135a-3p, miR-103a-3p, miR-7-1-3p, miR-107, miR-629-5p, and miR-362-5p).

**Figure 5:**
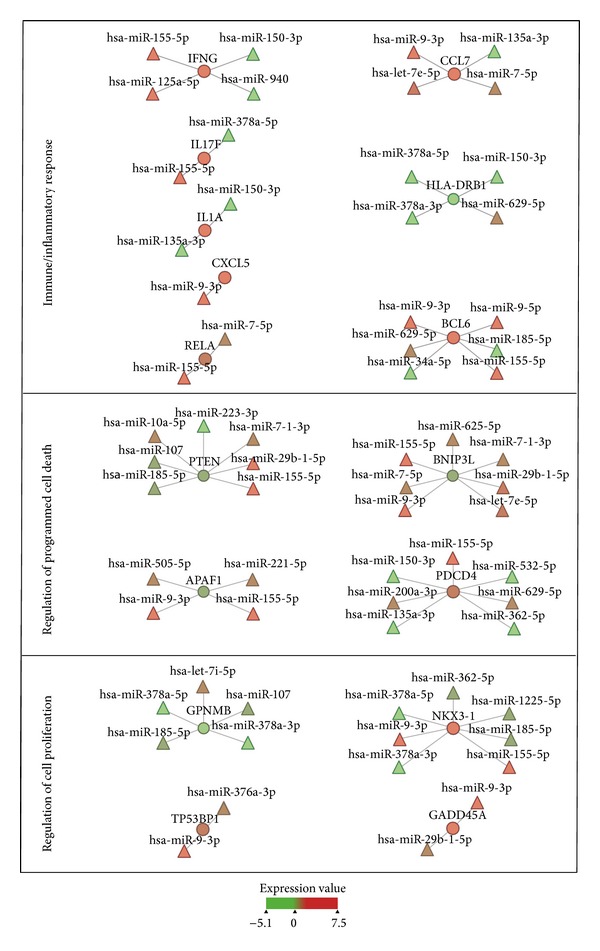
Cytoscape visualization of miRNA-mRNA correlations in PBLs incubated 24 h in MMG. Relationships between miRNAs and correlated target genes involved in “immune/inflammatory response” (IFNG, CCL7, IL17F, HLA-DRB1, IL1A, CXCL5, RELA, and BCL6), “regulation of programmed cell death” (PTEN, BNIP3L, APAF1, and PDCD4), and “regulation of cell proliferation” (GPNMB, NKX3-1, TP53BP1, and GADD45A). Circles represent transcripts and triangles represent miRNAs; the expression levels of each feature are represented as color scale.

By using Cytoscape [60], we visualized the functional interactions between miRNAs whose expression levels changed the most in PBLs incubated in MMG, such as miR-9-5p, miR-9-3p, miR-155-5p, miR-150-3p, and miR-378a-3p, and correlated target genes involved in GO categories of immune/inflammatory response, regulation of programmed cell death, and regulation of cell proliferation (Figure 5).

Our results show that miR-155-5p correlates with IFNG, IL17F, BCL6, and RELA involved in immune/inflammatory response, with PTEN, BNIP3L, APAF1, and PDCD4 involved in regulation of programmed cell death, and with NKX3-1 involved in regulation of cell proliferation; miR-150-3p correlates with immune-related genes (IFNG, IL1A, and HLA-DRB1) and with proapoptotic gene PDCD4; miR-9-3p correlates with genes regulating cell proliferation (NKX3-1, GADD45A, and TP53BP1), apoptosis (APAF1, BNIP3L), and immunity (CCL7, CXCL5, and BCL6). Among genes enriched within the three functional categories, miR-9-5p correlates with BCL6. miR-378a-3p is correlated with HLA-DRB1, GPNMB, and NKX3-1; the same transcripts, together with IL17F, are correlated also with miR-378a-5p.

**Figure 6:**
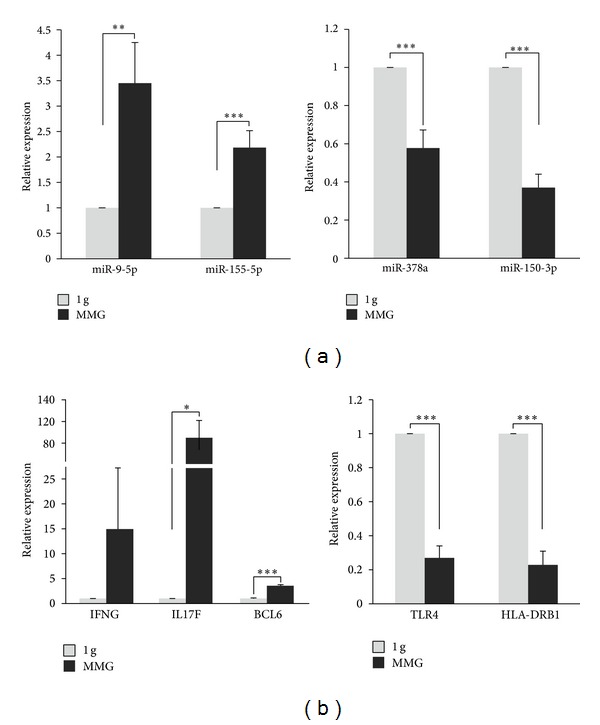
Microarray data validation by quantitative real-time PCR (qRT-PCR). Validation of microarray data by qRT-PCR in MMG-incubated versus 1 g incubated PBLs. The results are consistent with the cumulative microarray data of miRNAs (a) and mRNAs (b). Values (fold change, dark grey bars) are means ± S.E. of expression levels calculated as the log2 (MMG/1 g) on PBL samples from 4 to 6 different donors. The value “1” of control 1 g PBLs (light grey bars) is arbitrarily given when no change is observed (***P < 0.001, **P < 0.01, and *P < 0.05,* t*-test).

The microarray data from miRNA and gene expression profiling were validated by real-time qPCR experiments for four miRNAs (miR-9-5p, miR-155-5p, miR-378a, and miR-150-3p) and five mRNAs (IFNG, IL17F, BCL6, HLA-DRB1, and TLR4) whose expression level was significantly altered by MMG incubation (Figure 6). miR-9-5p and miR-155-5p, together with IFNG, IL17F, and BCL6 transcripts, were upregulated in MMG, whereas miR-378a and miR-150-3p together with HLA-DRB1 and TLR4 transcripts were downregulated in MMG.

### 3.4. Intraclass Integrated Analysis

Recently, Censi and colleagues [61] observed a significant increase in the number and strength of genes correlation under stress conditions, such as disease and environmental or physiological changes. To evaluate whether the stress induced by MMG increases the amount of correlation of the system with respect to 1 g control condition, we integrated mRNAs and miRNAs data separately for MMG and 1 g using MAGIA^2^. By comparing the two regulatory networks, we observed a similar number of interactions between MMG (190 interactions) and 1 g (218 interactions) (Figure S1), indicating that there was no significant connectivity enrichment under modeled microgravity. By contrast, Gene Ontology analysis performed on MMG- and 1 g-specific interactions reported 50 GO categories significantly enriched in only MMG condition (*P* < 0.2 after Benjamini corrections, Supplementary Table S7); 10 out of these were previously described in Table 1. In particular, the GO categories “regulation of cellular process” and “cell differentiation” were significantly affected by MMG. With intraclass analysis, the GO categories of immunity and cell death were not enriched in MMG probably because, in our study, the number of PBL samples available for such analysis was relatively small. On the whole, the intraclass analysis shows that modeled microgravity does not increase the general connectivity of miRNA-gene interaction network but rather increases the transcriptome plasticity with respect to 1 g gravity condition, as evidenced by the enriched GO categories.

### 3.5. *In Vitro* Validation of GO Analysis: Effects of MMG on Cell Proliferation and Apoptosis

**Figure 7:**
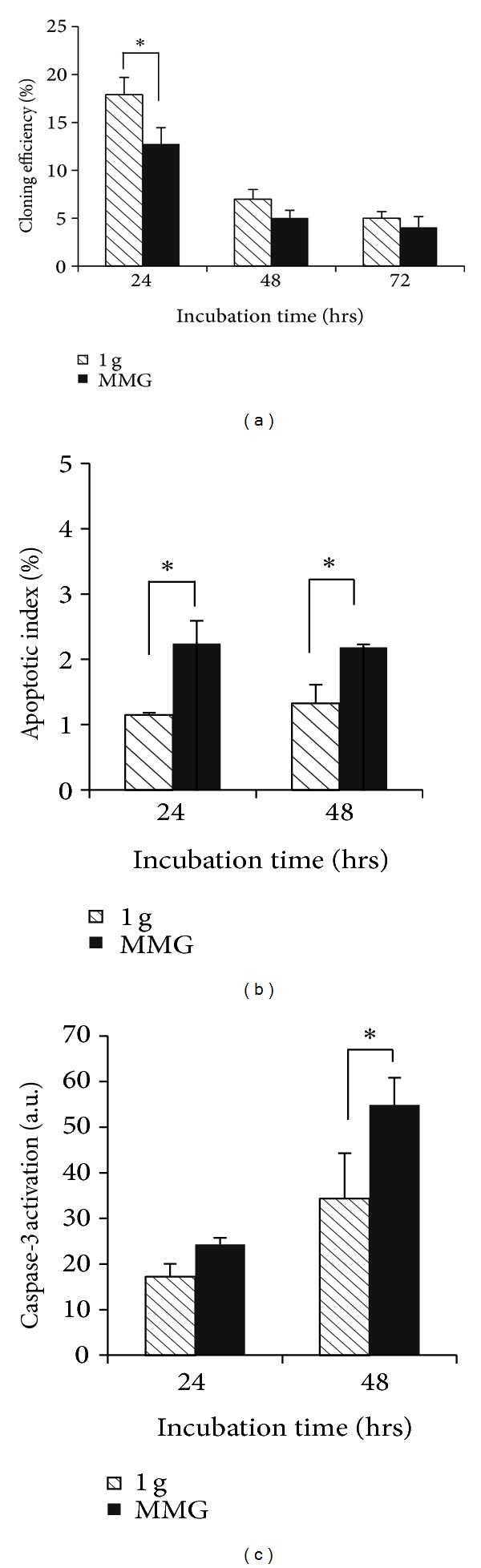
Cell proliferation and apoptosis induction in human PBLs incubated in MMG and in 1 g. (a) T-cell cloning assay performed at the end of 24 h, 48 h, and 72 h of incubation in the two gravity conditions. Data are means ± S.E. from thirteen independent experiments. (b) Apoptotic index at the end of incubation for 24 h and 48 h in MMG and 1 g determined by nuclear chromatin condensation with DAPI staining. (c) Caspase-3 activation at the end of 24 h and 48 h incubation in MMG and 1 g assessed by fluorimetric assay (a.u. arbitrary units). Data in (b) and (c) are means ± S.E. from 3-4 independent experiments (*P < 0.05,* t*-test).

Experimental assays were performed to validate the results obtained by bioinformatics analyses on the GO categories “regulation of cell proliferation” and “regulation of programmed cell death” associated with variations in miRNA expression under MMG. To measure cell proliferation, quiescent (*G*_0_) PBLs from the same donor were incubated for different times (24 h, 48 h, and 72 h) in 1 g and in MMG. At the end of incubation times, the colony forming ability has been determined by the T-cell cloning assay [44], in which cells were incubated in medium containing mitogen factors (i.e., PHA and IL2) to trigger their cell cycle entry. Our results showed that cloning efficiency (CE) decreased with time in both gravity conditions; however, MMG incubation affected the ability of PBLs to form colonies (*P* < 0.05 at 24 h, Figure 7(a)). To investigate whether MMG incubation increased the frequency of apoptotic cells, PBLs were scored for the presence of apoptotic bodies. Apoptotic index was very similar at 24 and 48 h and significantly higher in PBLs incubated in MMG than in 1 g (Figure 7(b), *P* < 0.05). In the same PBL samples, caspase-3 activation, assayed by the cleavage of the peptide substrate DEVD-AFC, increased significantly in PBLs incubated 48 h in MMG with respect to those in 1 g (Figure 7(c),  *P* < 0.05).

## 4. Discussion

In the present study, we evaluated the effects of modeled microgravity (MMG) on human PBLs by analyzing miRNA and gene expression profiles in comparison with PBLs cultured in Earth gravity condition (1 g). Our results reported 42 differentially expressed miRNAs in PBLs cultured for 24 h in MMG with respect to 1 g, of which 14 (miR-34a-5p, miR-34b-5p, miR-663a, miR-135a-3p, miR-1225-5p, miR-940, miR-221-5p, miR-29b-1-5p, miR-10a-5p, let-7i-3p, miR-200a-3p, miR-7-5p, miR-7-1-3p, and miR-505-5p) were found altered also by *γ*-irradiation, as assessed in our previous study [47]. The most dysregulated miRNAs identified in the present work are the upregulated miR-9-5p, miR-9-3p, and miR-155-5p, and the downregulated ones are miR-150-3p and miR-378a-3p. Such miRNAs have been found altered in human tumors; in particular, miR-9 is an oncogenic miRNA overexpressed in mixed lineage leukemia- (MLL-) rearranged acute myeloid leukemia [62], in muscle-invasive bladder cancer [63], and in osteosarcoma cell lines [64]. miR-155 is commonly upregulated in hematological malignancies [65, 66] and has been linked to the development of breast, lung, and stomach tumors [67–70]. miR-150 is significantly downregulated in most cases of acute myeloid leukemia [71] and colorectal cancer [72]; in addition, miR-150 has an important role in normal hematopoiesis and its aberrant downregulation is a sensitive marker indicative of lymphocyte depletion and bone marrow damage [73]. miR-378 (actually annotated as miR-378a) is significantly downregulated in colorectal cancer [74], in cutaneous squamous cell carcinoma [75], and in renal cell carcinoma [76]. The effects of microgravity on miRNA expression profile are currently reported in only one study carried out in human lymphoblastoid TK6 cells incubated under simulated microgravity for 72 h [77]. Among the dysregulated miRNAs, only two were common to our data, miR-150 and miR-34a, although the direction and intensity of fold change were different, demonstrating the cell type specific signature of miRNA profile.

miRNAs modulate gene expression by interacting with the 3′UTR of target genes, and since a single miRNA could have hundreds to thousands of predicted target genes [25], it is difficult to determine the true target regulated by the miRNA which affects a biological function. Moreover, binding of multiple miRNAs to one target could further increase the complexity of target prediction. The identification of miRNA target genes is usually performed by bioinformatic prediction algorithms based on (i) sequence similarity search, possibly considering target site evolutionary conservation, and (ii) thermodynamic stability. However, it is known that the results of target prediction algorithms are characterized by very low specificity [78]. For this purpose, the integration of target predictions with miRNA and gene expression profiles has been recently proposed to improve the detection of functional miRNA target relationships [79, 80]. Therefore, to identify the most likely target genes of miRNAs differentially expressed in MMG, we defined gene expression signature on the same samples of PBLs assayed for miRNA profiling; then, we integrated expression profiles from both miRNAs and mRNAs with* in silico* target predictions to reduce the number of false positives and increase the number of biologically relevant targets [81–83]. Our results of gene expression profiling reported the downregulation of multiple genes in MMG (71%), in accordance with previous findings in activated human T lymphocytes incubated for 24 h in simulated microgravity [21]. Moreover, we found that about 20% of genes responded to MMG by more than 2-fold change in expression level and twenty genes showed a ≥16-fold change in expression. Most of these top dysregulated genes were immune-related, such as those codifying for inflammatory cytokines (CCL1, CCL7, CXCL5, CXCL11, and IL1A) and for proteins with a role in immunoregulatory functions (IFNG, TNIP3, TREM1, APOC1, FCN1, FCN2, and CPVL) (Supplementary Table S3). Biological pathways enriched in PBLs exposed to MMG were mainly involved in immunity (Figure 2(b)), including adaptive immune system response (i.e., PD-1 signaling, phosphorylation of CD3 and TCR zeta chains, translocation of ZAP-70 to immunological synapse MHC class II antigen presentation, TCR signaling, and costimulation by the CD28 family), innate immune system response (i.e., Toll Receptor Cascades), and cytokine signaling in immune system (i.e., interferon gamma signaling) (Supplementary Table S4). All these pathways included ten downregulated genes codifying for MHC molecules class II (HLA-DPA1, HLA-DPB1, HLA-DQA1, HLA-DQA2, HLA-DQB1, HLA-DRA, HLA-DRB1, HLA-DRB3, HLA-DRB4, and HLA-DRB5), which are expressed in antigen presenting cells (APC) and play a central role in the immune system by presenting peptides derived from extracellular proteins [84]. Therefore, the downregulation of these genes suggests that the display of antigens at the cell surface of APC may be disturbed by gravity reduction, affecting the efficiency of immune response as observed in astronauts during spaceflight and immediately afterwards [85–87]. Moreover, our data are in accordance with the inhibition of immediate early genes in T-cell activation observed in space microgravity [14] and with alterations of gene expression in human activated T-cells incubated in modeled microgravity, including the downregulation of HLA-DRA gene [21].

By integrating the transcriptome and microRNAome, we detected significant miRNA-mRNA relationships under MMG. Since miRNAs act prevalently through target degradation, expression profiles of miRNAs and target genes are generally expected to be inversely correlated. Nevertheless, since miRNA activity is part of complex regulatory networks and gene expression profiles are the result of different levels of regulation, also positive correlation (i.e., upregulated miRNA/upregulated mRNA or downregulated miRNA/downregulated mRNA) is expected. Indeed, in an* in vivo* mouse model, the activated expression of miRNAs has been shown to correlate with activated expression of mRNAs rather than with mRNA downregulation [88]. Gene Ontology (GO) analysis conducted on the significantly correlated miRNA-mRNA pairs evidenced the biological categories significantly overrepresented in MMG (Table 1). Many GO terms of immune response were enriched, such as “innate immune response,” “inflammatory response,” “regulation of cytokine production,” “positive regulation of immune system process,” and “response to bacterium.” Notably, the most dysregulated miRNAs detected in the present study, miR-378a-3p, miR-150-3p, miR-155-5p, miR-9-3p, and miR-9-5p, are significantly correlated with immune-related genes. In particular, miR-378a-3p is positively correlated with transcripts of MHC molecules class II such as HLA-DRB1 (Figure 5) and HLA-DOA, HLA-DRB5, and HLA-DQA2 (not shown). miR-150-3p is negatively correlated with HLA-DRB1, IFNG, and IL1Atranscripts, whereas miR-155-5p is positively correlated with IFNG and IL17F and negatively correlated with RELA and BCL6 (Figure 5). IL1A, IL17F, and IFN-*γ* are proinflammatory cytokines acting during the immune response; IFN-*γ* is a soluble cytokine having broader roles in activation of immune responses, in part through upregulating transcription of genes involved in antigen processing/presentation, in cell cycle regulation and apoptosis, and its correlation with miR-155 has been recently validated [89]. BCL6, which encodes a nuclear transcriptional repressor, has a role not only in regulation of lymphocyte function, but also in cell survival and differentiation. Similarly, the pleiotropic transcription factor RELA has a role in immune biological process and it is also involved in cell growth and apoptosis. Besides miR-155-5p, BCL6 is correlated with four miRNAs including miR-9 (3p and 5p); in addition, miR-9-3p is positively correlated with CCL7 and CXCL5 (Figure 5). Recent evidences show that miR-9 is highly involved in immunity and inflammatory diseases [90–92] by enhancing IFN-*γ* production in activated human CD4(+) T-cells [91]. Moreover, Gao et al. [90] have shown that miR-9 disturbs the display of antigens at the cell surface by suppressing the expression of MHC class I gene transcription.

Together with the categories of immune response, GO analysis reported that also the categories of regulation of cell proliferation and regulation of programmed cell death were significantly enriched in MMG, as previously reported in *γ*-irradiated PBLs [47]. Interestingly, such categories were not enriched from pathway analysis conducted on transcriptome and the reason could be that integrated analysis of miRNAs and mRNAs expression profiles evidences the posttranscriptional effect mediated by miRNAs on gene expression. Among genes involved in cell proliferation, the transcription factor NKX3-1 (2.4-fold upregulated), which mediates non-cell autonomous regulation of gene expression and inhibits cell proliferation, is correlated with miR-9-3p, miR-155-5p, miR-378a-3p, and miR-378-5p (Figure 5). TP53BP1 (1.4-fold upregulated), encoding for a chromatin-associated factor involved in cell cycle checkpoint and growth, is correlated with miR-9-3p. GADD45A (1.5-fold upregulated), regulating cell cycle arrest, DNA repair, cell survival, senescence, and apoptosis, is also correlated with miR-9-3p. GPNMB (32-fold downregulated), expressed in a wide array of normal tissues, such as bone, hematopoietic system, and skin, where it influences cell proliferation, adhesion, differentiation, and synthesis of extracellular matrix proteins [93], is targeted by five miRNAs including miR-378a-3p. Among miRNA-correlated genes involved in apoptosis, we identified PDCD4 (proapoptotic, 1.4-fold upregulated) and found out that it is correlated with seven miRNAs, including miR-155-5p and miR-150-3p; BNIP3L (proapoptotic, 1.5-fold downregulated) also correlated with seven miRNAs, including miR-155-5p and miR-9-3p; APAF1 (proapoptotic, 1.3-fold downregulated) correlated with four miRNAs including miR-155-5p and miR-9-3p; and PTEN (proapoptotic) correlated with seven miRNAs including miR-155-5p. Notably, many transcripts (i.e., BCL6, PTEN, BNIP3L, PDCD4, NKX3-1, and GPNMB) are targeted by multiple miRNAs, indicating a pleiotropic effect in gene regulation by coexpressed endogenous miRNAs in MMG. Moreover, our results suggest that under MMG condition a small group of miRNAs regulates transcriptome by modulating the same transcripts within one pathway.

To validate the results of Gene Ontology analysis, we evaluated whether the GO categories “regulation of cell proliferation” and “regulation of programmed cell death” were affected by MMG incubation by performing biological assays of T-cell cloning and apoptosis induction. Our results show that cloning ability of PBLs was lower after 24 h incubation in MMG than in 1 g, in accordance with the suppression of proliferative response of human lymphocytes to mitogenic stimulation in microgravity [94, 95]. The ability to originate clones in PBLs incubated for 48h and 72 h decreased in both gravity conditions probably because the longer *G*_0_-phase condition experienced by PBLs affected their responsiveness to enter into cell cycle. The antiproliferative effect of microgravity has been recently reported also in human thyroid cancer cells [96] and in human lung adenocarcinoma cells [97]. Our results of apoptosis induction demonstrated that both the formation of apoptotic bodies activation and caspase-3 activation increased significantly in PBLs incubated in MMG than in 1 g. The activation of apoptotic process seems related to the overexpression of PDCD4 and RELA rather than to the underexpression of BNIP3L and APAF1, indicating the existence of complex regulatory networks between miRNAs and mRNAs that occur at different levels of regulation. IFN-*γ*, besides having an important role in activating innate and adaptive immune responses, plays important roles in inhibiting cell proliferation and inducing apoptosis. Its overexpression in MMG, mediated by miR-9-3p and miR-155-5p, could thus mediate the antiproliferative effect and the apoptosis induction. In addition, the correlation between miR-9-3p and TP53BP1 could explain the clonogenicity decrease and apoptosis increase in PBLs incubated in MMG. Indeed, overexpression of TP53BP1 has been demonstrated to decrease the clonogenicity and induce apoptosis in ovarian cancer cells [98].

## 5. Conclusions

Our results show that MMG leads to changes in expression level of a considerable fraction of microRNAome and transcriptome in human PBLs. miRNAs differentially expressed in MMG are correlated with immune/inflammatory-related genes, as IFNG, accordingly with the important role of miRNAs in immune function regulation. Since inflammation works as a tumor-promoting agent, the abnormal expression of such miRNAs under microgravity condition could influence the carcinogenic process by affecting cancer cell immune escape. Moreover, miRNAs mostly dysregulated in MMG, such as miR-9, miR-155, and miR-150, are oncogenic, suggesting that their abnormal expression can influence the carcinogenic process. The results of miRNA-mRNA integration analysis demonstrate that MMG increases the transcriptome plasticity compared with 1 g condition and that categories of regulation of cell proliferation and programmed cell death are affected by MMG, as confirmed by* in vitro* experimental validation. Taken together,our results of high-throughput expression analysis and miRNA-mRNA integration analysis give new insight into the complex genetic mechanisms of cell response to stress environment under reduced gravity.

## Supplementary Material

Supplementary Table S1: reports the list of miRNAs differentially expressed in PBLs incubated 24h in modeled microgravity (MMG). The table includes miRNA ID and the expression value of each PBL sample (12 donors, A-P) expressed as log2 (MMG/1g).
Supplementary Table S2: reports the list of differentially expressed genes in PBLs incubated 24h in in modeled microgravity (MMG).The expression level (FC, fold-change) of each gene is expressed as log2 (MMG/1g).
Supplementary Table S3: reports the list of differentially expressed genes showing a fold change greater than 16.0 in PBLs incubated in in modeled microgravity (MMG).
Supplementary Table S4: reports pathways significantly enriched in PBLs incubated in modeled microgravity (MMG). Pathway analysis has been performed by using hypergeometric test on Reactome Pathways as implemented in Graphite web, considering significant those categories with a FDR < 0.1.
Supplementary Table S5: reports miRNA-correlated target genes in PBLs incubated in in modeled microgravity (MMG).The correlation analyses were carried out with MAGIA2 software, by microRNA Pearson prediction analysis.
Supplementary Table S6: reports the complete list of GO terms in PBLs incubated in modeled microgravity (MMG).
Supplementary Table S7: reports GO terms of biological processes affected by modeled microgravity (MMG) identified from intra-class analysis. Supplementary Figure S1 shows the results of Intra-class analysis in PBLs incubated in MMG and 1g. (a) The Venn diagram shows the number of intra-class interactions in MMG, in 1g, and shared by the two gravity conditions. (b) Regulatory miRNA-gene networks relative to MMG- and 1g-specific interactions. Circles and triangles represent respectively genes and miRNAs; the expression levels of each features are represented as color scale.

## Figures and Tables

**Table 1:** Selected GO terms of biological processes significantly affected by microgravity. The complete list of GO terms can be found in Supplementary Table S6.

GO:ID	Term	Count	*P* value	Fold enrichment	FDR
GO:0045087	Innate immune response	25	2,42 × 10^−5^	2,566978193	0,040412626
GO:0009966	Regulation of signal transduction	88	1,67 × 10^−4^	1,471577879	0,27973741
GO:0048468	Cell development	62	1,71 × 10^−4^	1,610338849	0,285253218
GO:0045595	Regulation of cell differentiation	54	2,68 × 10^−4^	1,648	0,448057857
GO:0006954	Inflammatory response	41	3,23 × 10^−4^	1,787513228	0,539044451
GO:0080134	Regulation of response to stress	37	5,43 × 10^−4^	1,806696296	0,90478009
GO:0010646	Regulation of cell communication	95	8,22 × 10^−4^	1,380599647	1,366092587
GO:0048513	Organ development	139	8,22 × 10^−4^	1,290910116	1,366713032
GO:0042060	Wound healing	26	8,38 × 10^−4^	2,025910165	1,393334889
GO:0007165	Signal transduction	198	0,001398603	1,211224944	2,313943814
GO:0001817	Regulation of cytokine production	27	0,001406003	1,926233766	2,326052387
GO:0048514	Blood vessel morphogenesis	26	0,001709187	1,93009009	2,820918727
GO:0001817	Regulation of cytokine production	15	0,001406003	1,926233766	2,326052387
GO:0007399	Nervous system development	88	0,002097026	1,359812471	3,450524099
GO:0022603	Regulation of anatomical structure morphogenesis	28	0,002408369	1,831111111	3,953172097
GO:0048870	Cell motility	33	0,002838826	1,710188679	4,64407324
GO:0048522	Positive regulation of cellular process	159	0,003551259	1,221594406	5,777298149
GO:0002684	Positive regulation of immune system process	31	0,003855111	1,711490787	6,256755641
GO:0050865	Regulation of cell activation	26	0,003880839	1,819447983	6,297247375
GO:0051174	Regulation of phosphorus metabolic process	51	0,003964604	1,486259947	6,428964883
GO:0001944	Vasculature development	28	0,003981783	1,767969349	6,455956993
GO:0043066	Negative regulation of apoptosis	41	0,004040803	1,56952381	6,548633436
GO:0010941	Regulation of cell death	83	0,004115909	1,341019608	6,666445839
GO:0009617	Response to bacterium	22	0,004991824	1,9032021	8,030143455
GO:0043067	Regulation of programmed cell death	82	0,00552374	1,328770895	8,849108723
GO:0008285	Negative regulation of cell proliferation	40	0,00576007	1,54741784	9,210770389
GO:0010557	Positive regulation of macromolecule biosynthetic process	62	0,006543793	1,39014966	10,4004889
GO:0016477	Cell migration	30	0,006706505	1,664646465	10,64564555
GO:0042127	Regulation of cell proliferation	71	0,009848433	1,331149033	15,2576907
GO:0048523	Negative regulation of cellular process	139	0,012514364	1,19870225	18,9945349
