# Supplementary figures and images for: Integration Analysis of MicroRNA and mRNA Expression Profiles in Human Peripheral Blood Lymphocytes Cultured in Modeled Microgravity
Source: Biomed Res Int. 2014 Jun 23;2014:296747. doi: 10.1155/2014/296747 (PMC4090438; doi:10.1155/2014/296747)

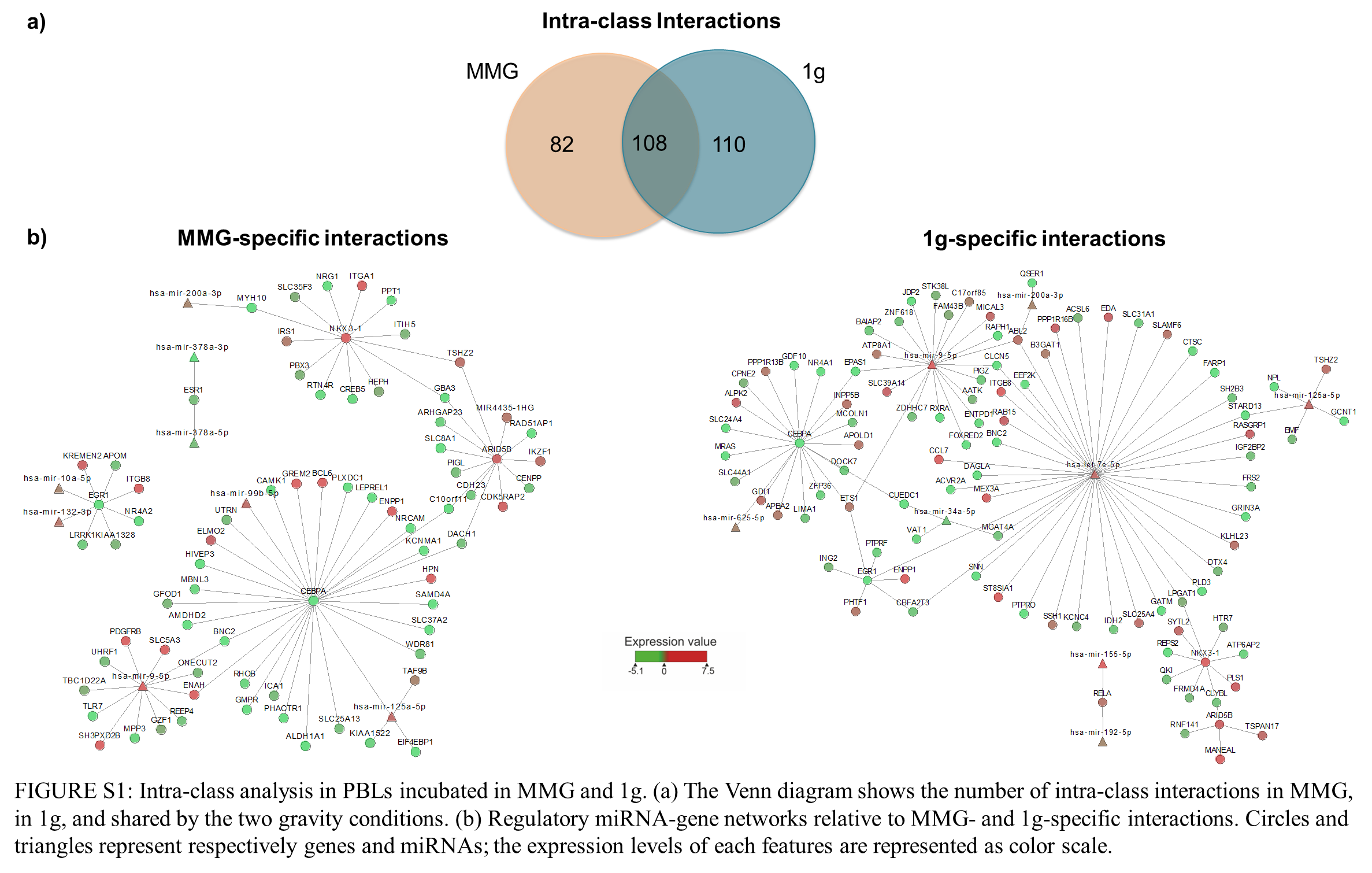

Supplement: Supplementary file 1 — Supplementary Table S1: reports the list of miRNAs differentially expressed in PBLs incubated 24h in modeled microgravity (MMG). The table includes miRNA ID and the expression value of each PBL sample (12 donors, A-P) expressed as log2 (MMG/1g). Supplementary Table S2: reports the list of differentially expressed genes in PBLs incubated 24h in in modeled microgravity (MMG).The expression level (FC, fold-change) of each gene is expressed as log2 (MMG/1g). Supplementary Table S3: reports the list of differentially expressed genes showing a fold change greater than 16.0 in PBLs incubated in in modeled microgravity (MMG). Supplementary Table S4: reports pathways significantly enriched in PBLs incubated in modeled microgravity (MMG). Pathway analysis has been performed by using hypergeometric test on Reactome Pathways as implemented in Graphite web, considering significant those categories with a FDR < 0.1. Supplementary Table S5: reports miRNA-correlated target genes in PBLs incubated in in modeled microgravity (MMG).The correlation analyses were carried out with MAGIA2 software, by microRNA Pearson prediction analysis. Supplementary Table S6: reports the complete list of GO terms in PBLs incubated in modeled microgravity (MMG). Supplementary Table S7: reports GO terms of biological processes affected by modeled microgravity (MMG) identified from intra-class analysis. Supplementary Figure S1 shows the results of Intra-class analysis in PBLs incubated in MMG and 1g. (a) The Venn diagram shows the number of intra-class interactions in MMG, in 1g, and shared by the two gravity conditions. (b) Regulatory miRNA-gene networks relative to MMG- and 1g-specific interactions. Circles and triangles represent respectively genes and miRNAs; the expression levels of each features are represented as color scale. [file 296747.f1.zip › Figure S1.tif]
